# Amniotic Membrane Grafts for the Prevention of Esophageal Stricture after Circumferential Endoscopic Submucosal Dissection

**DOI:** 10.1371/journal.pone.0100236

**Published:** 2014-07-03

**Authors:** Maximilien Barret, Carlos Alberto Pratico, Marine Camus, Frédéric Beuvon, Mohamed Jarraya, Carole Nicco, Luigi Mangialavori, Stanislas Chaussade, Frédéric Batteux, Frédéric Prat

**Affiliations:** 1 Department of Gastroenterology, Cochin Hospital, Paris, France; 2 Faculté Paris Descartes, Paris, France; 3 Department of Pathology, Cochin Hospital, Paris, France; 4 Human Tissue Bank, Saint-Louis Hospital, Paris, France; 5 Department of Immunology and EA 1833, Cochin Hospital, Paris, France; Shiga University of Medical science, Japan

## Abstract

**Background and Aims:**

The prevention of esophageal strictures following circumferential mucosal resection remains a major clinical challenge. Human amniotic membrane (AM) is an easily available material, which is widely used in ophthalmology due to its wound healing, anti-inflammatory and anti-fibrotic properties. We studied the effect of AM grafts in the prevention of esophageal stricture after endoscopic submucosal dissection (ESD) in a swine model.

**Animals and Methods:**

In this prospective, randomized controlled trial, 20 swine underwent a 5 cm-long circumferential ESD of the lower esophagus. In the AM Group (n = 10), amniotic membrane grafts were placed on esophageal stents; a subgroup of 5 swine (AM 1 group) was sacrificed on day 14, whereas the other 5 animals (AM 2 group) were kept alive. The esophageal stent (ES) group (n = 5) had ES placement alone after ESD. Another 5 animals served as a control group with only ESD.

**Results:**

The prevalence of symptomatic strictures at day 14 was significantly reduced in the AM group and ES groups vs. the control group (33%, 40% and 100%, respectively, p = 0.03); mean esophageal diameter was 5.8±3.6 mm, 6.8±3.3 mm, and 2.6±1.7 mm for AM, ES, and control groups, respectively. Median (range) esophageal fibrosis thickness was 0.87 mm (0.78–1.72), 1.19 mm (0.28–1.95), and 1.65 mm (0.7–1.79) for AM 1, ES, and control groups, respectively. All animals had developed esophageal strictures by day 35.

**Conclusions:**

The anti-fibrotic effect of AM on esophageal wound healing after ESD delayed the development of esophageal stricture in our model. However, this benefit was of limited duration in the conditions of our study.

## Introduction

Endoscopic therapy has become a valid therapeutic option in the management of Barrett's esophagus (BE) complicated with high grade dysplasia or early adenocarcinoma [1]. The treatment can be limited to the resection or ablation of neoplasia, but can also be aimed at eradicating the entire metaplastic mucosa [1]. Hence, techniques allowing circumferential treatment of the esophageal mucosa are required. To achieve this goal, either mucosal resection (using EMR or submucosal dissection) or radiofrequency ablation, or a combination of methods may be used [2]. Radiofrequency ablation has a number of limitations: it is restricted to flat lesions, does not allow for histological analysis of ablated tissues, lacks long-term (>5 years) efficacy data [3], and subsequent follow-up appears to be difficult, with cases of buried metaplasia, high grade dysplasia and adenocarcinoma reported after complete BE eradication [4]. Endoscopic mucosal resection is feasible, even in circumferential BE [5], but requires piece-meal resection, with suboptimal histological analysis and a risk of residual dysplastic mucosa; furthermore, circumferential mucosal resection leads to severe esophageal strictures in around 37% to 92% of cases [5], [6], [7], as previously reported in mucosal defects involving more than three-quarters of the esophageal circumference [8]. Circumferential endoscopic submucosal dissection (CESD) could be the optimal therapeutic option in the setting of complicated circumferential BE, but is time-consuming, and results in severe esophageal strictures in up to 75% of cases in humans [9], [10]. To address the first problem, we developed a CESD technique on a swine model allowing for safe *en bloc* resection of 5 cm-long cylinders of esophageal mucosa and submucosa in less than an hour [11]. However, this procedure led to a 100% stricture rate by day 14. Thus, the question of post-endoscopic esophageal stricture prevention remains to be addressed.

Human amniotic membrane (AM) consists of an avascular stroma and a monostratified cylinder cell epithelium expressing very few histocompatibility antigens [12]. Therefore, it is not likely to induce immune rejection. AM promotes epithelial growth through the secretion of epithelial growth factors (EGF, KGF and HGF) [13] and by playing the role of a scaffold supporting epithelial cell proliferation and migration. Secondly, AM has anti-angiogenic properties, possibly associated with the presence of thrombospondin 1 and of potent neoangiogenesis inhibitors (tissue inhibitors of metalloproteinases or TIMPs) [14], both in the epithelium and the stroma of AM [15], [16]. Thirdly, AM has anti-inflammatory and anti-fibrotic potential, related to the presence in the AM of all four types of TIMPs, interleukin 1 receptor antagonist, and interleukin 10 [15]. AM inhibits TGFβ synthesis and myofibroblastic differentiation, which strongly supports the hypothesis of AM possessing anti-fibrotic properties [15], [17], [18]. Furthermore, as reported in the setting of preeclampsia, the presence of lactoferrin in AM can also result in its anti-oxidant properties [19]. AM has been tested in various medical fields to promote wound healing, especially in burns or skin ulcers [20], and is currently used in ophthalmology for ocular surface reconstruction, especially in the treatment of corneal ulcers [21]. It may be used as a graft, with the stroma applied on the wound, or as a patch, with the epithelial layer placed towards the wounded tissue, in order to temporarily cover the wound and deliver the growth factors contained within the epithelium directly to the lesion [16].

Based on this rationale, we conducted a randomized controlled trial on a swine model in order to assess the role of AM grafts in the prevention of esophageal stricture development after CESD.

## Animals and Methods

### Animal experiments

The experimental protocol received approval from the Institutional Review Board of the Paris School of Surgery (Ecole de Chirurgie de l'Assistance Publique - Hôpitaux de Paris, Paris, France), and experiments were performed according to the Standard Guidelines of the French Ministry of Agriculture, which regulates animal research in France. All efforts were made to minimize suffering.

Twenty 30–35 kg swine originating from the same farm were used in the study. The animals were randomly allocated to groups as follows: treatment by AM applied using an esophageal stent (n = 10), esophageal stent (ES) placement alone (n = 5) or control group (n = 5). The animals were accommodated at our facility 48 hours before the procedure took place. Endoscopies were performed under general anesthesia. All animals were prepared for anesthesia with a 12-hour diet and were administered an intramuscular injection of 10 mg/kg ketamine and 2 mg/kg azaperone, 30 minutes before induction. Following induction with 8 mg/kg intravenous 1% propofol and endotracheal intubation, anesthesia was maintained through the inhalation of 1% to 2% isoflurane. Animals received an intravenous infusion of 10 mg/kg/h crystalloid solution.

### Study design and group allocation

Since esophageal strictures are known to occur around the 14^th^ postoperative day in our model [22], a subgroup of 5 animals in the AM group was randomly selected at inclusion (AM1 group, n = 5), to be sacrificed at day 14, in order to obtain histological data to be compared to the control group at the same time interval from ESD. The rest of the AM group (AM2 group, n = 5) was followed-up.

### ESD procedure

Upper gastrointestinal endoscopies were performed using a standard gastroscope (Fujinon EG450D, Fujinon, Sataima, Japan), through an overtube. A senior endoscopist carried out or directly supervised the procedure at all times. Circumferential submucosal endoscopic dissection of the distal third of the esophagus was performed as previously described [11], [22]. Briefly, two circumferential incisions reaching the superficial submucosal layer were made after submucosal injection of indigo carmine-stained saline; these two incisions defined a mucosal cylinder of 5 cm in the lower third of the esophagus, 5 cm above the gastroesophageal junction. The cylinder was removed by blunt submucosal dissection, using a distal transparent attachment as used for EMR and ESD (ST HOOD DH-19 GR, Fujinon, Sataima, Japan), which allowed cleavage of the mucosa and superficial submucosa from the underlying submucosa by gently scraping the esophageal wall in a back and forth motion, from the proximal to the distal incision. The resected mucosal cylinder was then retrieved using the endoscope's suction channel.

### Amniotic membrane application

AM grafts that were cryopreserved at −80°C were obtained from the Human Tissue Bank in the St Louis Hospital, Paris, France, after standardized processing [21], and were brought to the Paris Surgery School in dry ice. After 30 minutes of defrosting at room temperature, AM grafts of approximately 16 cm^2^ were stained in a sterile saline and indigo carmine bath to allow for easier identification after application.

We conducted preliminary experiments to determine the best modality for AM application. The over the scope application of AM, using clips to attach the AM to the esophageal wall, or the through the scope AM application, using a dilation balloon (as described with porcine small intestinal submucosa by Nieponice et al. [23]), were not satisfactory, because of the fragility of AM. AM application using a self-expanding plastic esophageal stent was found to be the optimal technique. Stent removal was planned on day 7, in order to distinguish the role of AM and from the stent.

**Figure 1:**
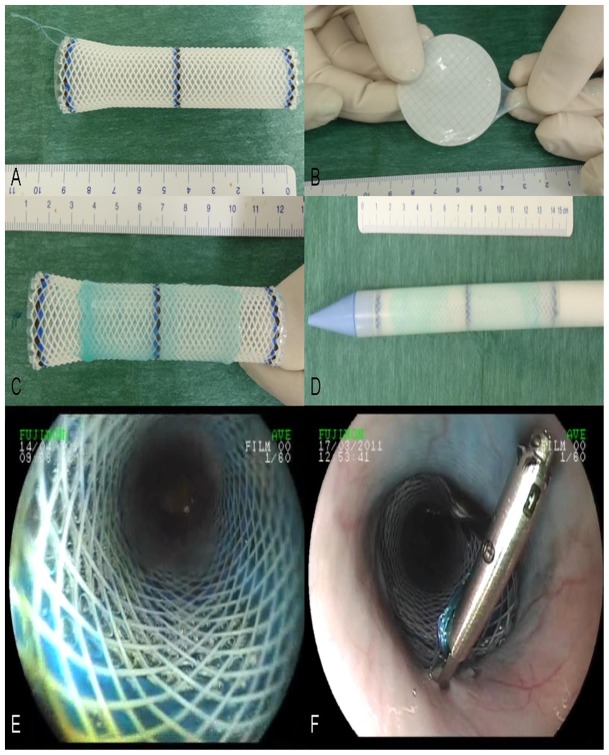
Application of amniotic membrane grafts on esophageal wounds. A: esophageal stent with attached non-absorbable suture; B: blue-stained amniotic graft on a nitrocellulose sheet after defrosting; C: amniotic membrane apposition on the external side of a Polyflex stent; D: esophageal stent coated with amniotic membrane graft loaded in the stent catheter; E: endoscopic view of the coated esophageal stent in the esophagus; F: esophageal stent clipped to the esophageal wall using the suture.

After ESD, one to two (depending on the size of the graft) AM grafts were wrapped around extractible esophageal stents with a diameter of 16–20 mm and a length of 9 cm (Polyflex, Boston Scientific, Natick, MA). The epithelial side was placed towards the esophageal wall, and attention was paid to the fact that the entire wound area had to be covered. A non-resorbable surgical suture was tied at the proximal end of the stent (Mersutures 1, Ethicon, Sommerville, NJ) to facilitate fixation to the esophageal wall and repositioning if necessary. The stent was then loaded and placed under fluoroscopic guidance. AM graft placement is shown in figure 1. Endoscopic control of stent placement was performed, and was attached to the esophageal wall by a clip (Resolution, Boston Scientific, Natick, MA) through the suture to prevent stent migration.

### Postoperative care and follow-up

A seven-day course of omeprazole 40 mg/day and broad spectrum antibiotics was administered: 10 mg/kg extended release benzathine benzylpenicillin G and penicillin procain (Duphapen, Pfizer, NY, NY) for the first four days followed by 1 g/day amoxicillin (Clamoxyl, GSK, Brentford, UK) for three days. The animals were given a liquid diet following each procedure, and were gradually introduced to a solid food diet. Follow-up included daily visits, weekly veterinary examinations, and esophagogastroscopy. Clinical data including behavior, quantitative and qualitative assessment of food intake and the occurrence of regurgitation or vomiting were collected on a weekly basis. The width of the esophageal lumen in the operated area was measured using the open jaws of biopsy forceps. Esophageal stricture was diagnosed when the esophageal lumen was narrower than the threshold of 9 mm, i.e. when the 9 mm endoscope could not pass through the esophageal lumen and reach the stomach. Symptomatic esophageal stricture was defined by the association of a strictured esophagus diagnosed by endoscopic examination (with an esophageal lumen narrower than 9 mm), and the presence of associated symptoms, such as anorexia, vomiting or regurgitation.

When a symptomatic esophageal stricture (confirmed by endoscopy) occurred, animals were euthanized with a 100 mg/kg intravenous injection of pentobarbital (Dolethal, Vétoquinol, Paris, France), and underwent necropsy with *en bloc* esophagectomy. Gross morphology analysis was performed, esophageal samples from strictured and healthy zones were taken and frozen at −80°C, and the remaining esophagus was fixed in 10% buffered formalin. The same procedure was applied to the animals in the AM 1 group, which were sacrificed on day 14 after ESD.

### Histological analysis

After gross morphological examination of the size, weight, and thickness of the esophageal wall, esophageal samples from healthy and wounded areas were taken and frozen at −80°C. Specimens were fixed in 10% buffered formalin, embedded in paraffin, and processed into 5 µm-thick sections. Slides were then stained with hematoxylin eosin and saffron (HES), and Masson's trichrome, as part of the histological examination. Slides of interest were digitized by an NDPI Nanozoomer (Hamamatsu photonics, Hamamatsu City, Japan), and submitted to semi-quantitative analysis of fibrosis, granulation tissue, and inflammatory cell infiltrate by a senior pathologist expert in digestive pathology (FB). The following measurements were made on each digitized image of trichrome-stained slides: a) maximal thickness of fibrosis extending between the muscularis propria and the epithelium; b) maximal thickness of granulation tissue extending between the esophageal lumen and the submucosal fibrosis; and c) length of re-epithelialization, by measuring the neoepithelium characterized by round immature epithelial cells without native epithelial crest and chorionic papillae or parakeratosis. The inflammatory cell infiltrate was characterized as acute (predominance of polynuclear cells, high cell density) or chronic (predominance of lymphocytes or plasmocytes, low cell density). Immunohistochemistry on paraffin sections was performed with a rabbit polyclonal antibody to alpha smooth muscle actin (ab5694, Abcam, Cambridge, UK) and a goat polyclonal Secondary Antibody to Rabbit IgG - H&L (HRP) (ab97051, Abcam, Cambridge, UK), in order to assess the intensity of myofibroblastic reaction in the esophageal wall.

### Oxidative stress measurements

Frozen esophageal samples were defrosted, chopped and ground in ripa, and then centrifuged at 5000 rpm for 10 mn at room temperature. The supernatant was carefully removed, diluted and aliquoted, and the following oxidative stress measurements were performed: superoxide dismutase (SOD) cellular activity was assessed by tetrazolium nitroblue reduction technique, as described by Beauchamp and Fridovich [24], and read at 550 nm; catalase cellular activity was determined by ultraviolet spectroscopy at 450 nm, as described by Aebi [25]. Advanced oxidation proteic products were measured by spectrophotometry, according to Witko-Sarsat [26], using chloramine T, and 365 nm absorbance on a Fusion reader (Fusion, Perkin Elmer, Wellesley, MA, USA). Carbonyl derivatives associated with oxidative stress and circulating thioredoxin were measured using dinitrophenylhydrazine. Total glutathione dosage was assessed using the 5, 5′-dithiobis(nitro-2-benzoïc) or DNTB technique. Finally, nitrates were measured through the cadmium method. The activity of antioxidant enzymes and cellular content in oxidative stress metabolites was normalized with respect to the protein concentration of each sample (bovine serum albumin microbiuret assay, Pierce, Bezons, France).

### Statistical analysis

Statistical analysis was performed using GraphPad Prism (GraphPad Software, La Jolla, CA). Continuous data are expressed as median values and range or mean ± standard deviation, as appropriate, and compared with a non-parametric Kruskal-Wallis test, followed by a Dunn's multiple comparison test. Categorical data are expressed as percentages and compared with a Fisher's exact test. A p value <0.05 was considered to indicate statistical significance.

### End points

The primary end point was the prevalence of symptomatic esophageal stricture at day 14 following ESD.

Secondary endpoints were: incidence of esophageal stricture at any time, global clinical evaluation and food intake at day 14, diameter of the esophagus measured via endoscopy, width of fibrosis, thickness of inflammatory granulation tissue and length of re-epithelialization in the esophageal wall, intensity of myofibroblastic reaction on immunohistochemistry, and intensity of oxidative stress in the esophageal wall.

## Results

Twenty swine were included in the study. The median (range) length of the resected esophageal mucosa was 5 (3–8) cm at a median (range) distance of 5 (2–5) from the esophagogastric junction. The median (range) time needed to complete ESD was 31 (17–80) min. Five minor adverse events, including two spontaneously resolving submucosal hemorrhages (in the control group), two ESDs beyond the esophagogastric junction (one in the AM 2 and one in the ES group), and an asymptomatic breach of the muscularis propria (in the AM 2 group) were recorded.

**Figure 2:**
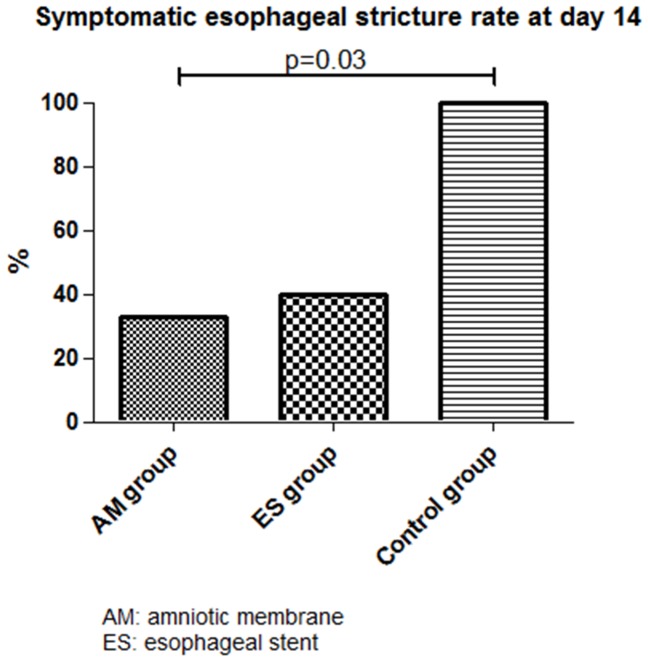
Symptomatic esophageal strictures rates at day 14.

A symptomatic esophageal stricture occurred in 33%, 40% and 100% of the swine in the AM, ES and control groups, respectively (p = 0.03 for AM vs. and ES vs. control group comparisons), as shown in figure 2. Clinical and endoscopic evaluation is described in table 1. Proximal or distal location of the esophageal stricture, as well as endoscopic features of the esophageal scar (re-epithelialization, erosions or ulcerations), were not significantly different among the 3 groups. Esophageal stents were still found in the esophagus in about 50% of the cases at day 7, equally distributed in all groups (3/5, 3/5, and 2/5 in AM 1, AM2, and ES group, respectively), and all of the remaining stents were removed at that time. The median (range) stricture-free survival was significantly different between the AM and control groups (22 (20–35) days vs.14 (14–14) days, p = 0.0005, HR = 49.4, 95%CI [5.44– 448.8]), and between the ES and control groups (21 (14–31) days vs.14 (14–14) days, p = 0.014, HR = 20.09, 95%CI [1.82–221.5]); however, the median stricture-free survival was not significantly different between the AM and ES groups (22 (20–35) days vs. 21 (14–31), p = 0.85, HR = 1.14, 95%CI [0.31–4.16]). By day 35 after ESD, all animals had developed a symptomatic esophageal stricture.

**Table 1:** Clinical and endoscopic evaluation on the 14^th^ postoperative day.

	AM group (n = 10)	ES alone group (n = 5)	Control group (n = 5)	p
Food intake n - (%)				
Diet				
Normal/doughy	9–90%	3–60%	1–20%	**0.008** (AM vs. Control group)
Liquid/no food intake	1–10%	2–40%	4–80%	
Tolerance				
Normal	8–80%	5–100%	1–20%	**0.048** (ES vs. Control group)
				**0.047** (AM vs. Control group)
Regurgitations/vomiting	2–20%	0	4–80%	
**Endoscopic evaluation** n - (%)				
Esophageal stricture	8–80%	4–80%	5–100%	NS
Symptomatic esophageal stricture	3–33%	4–40%	5–100%	**0.03** (AM vs. Control group)
Esophageal diameter*	5.8±3.6	6.8±3.3	2.6±1.7	NS

*: mean ± standard deviation, in mm;

NS: non significant.

AM: amniotic membrane.

ES: esophageal stent.

**Figure 3:**
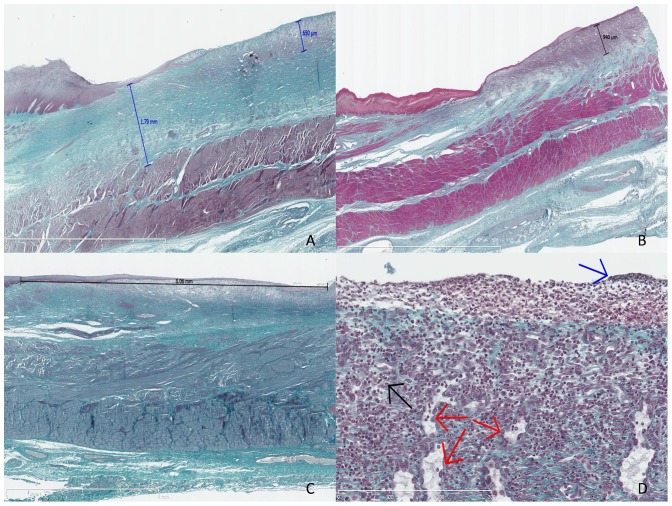
Histological analysis of the swine esophagus after Masson's trichrome staining. A: swine from the control group, sacrificed at day 14, with major fibrosis measured at 1.79 mm and thick granulation tissue measured at 0.65 mm, original magnification 12.5x; B: swine from the AM 1 group (amniotic membrane graft and early sacrifice scheduled at day 14), without esophageal stricture, with minimal fibrosis and mostly granulation tissue, measured at 0.94 mm, original magnification 10x; C: swine from the AM 2 group sacrificed at day 21, exhibiting major re-epithelialization measured at 8.06 mm, original magnification 15x; D: granulation tissue with features of acute inflammation, as observed in the early phase of esophageal wounds: high cell density, predominance of polynuclear cells (black arrow), fibrino-leucocytary network (blue arrow), and typical palissadic vascular growth (red arrows), original magnification 400x.

**Figure 4:**
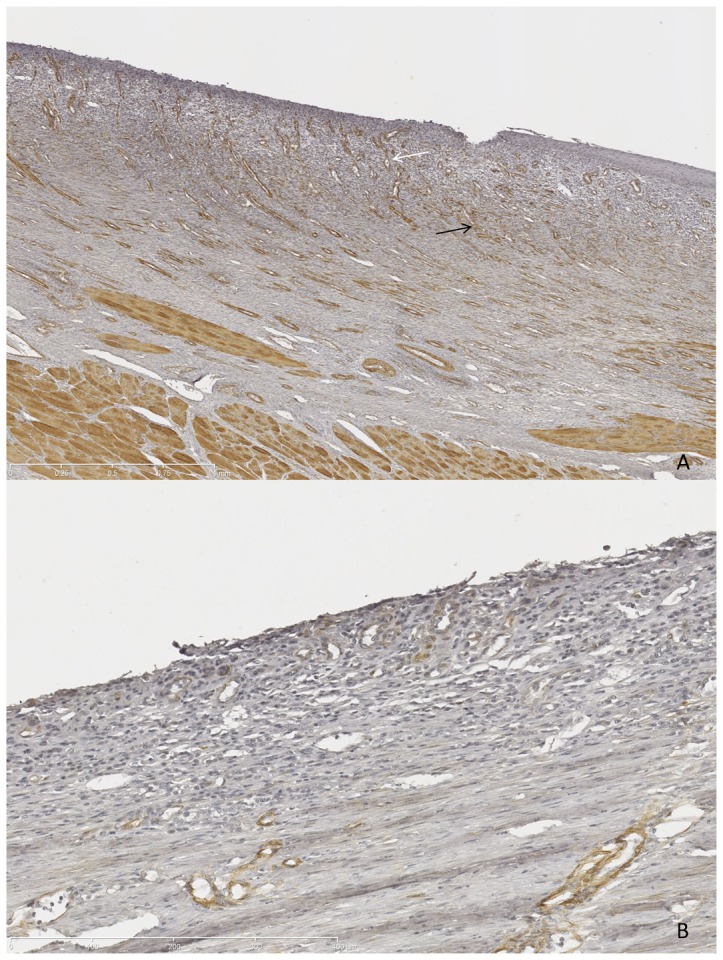
Immunohistochemistry staining with anti-αSMA antibody, original magnification ×200. Strong signal (brown spots) attesting high myofibroblastic activity (panel A, black arrow) and high vascular density (panel A, white arrow) in control (A); nearly absent signal in MA-treated swine (B).

Gross morphology showed whitish esophageal scars suggesting complete re-epithelialization in 90% of cases, and peri-esophageal lymphadenopathy. Histological study after Masson's trichrome staining allowed the first confirmation that the dissection had reached the submucosa in all cases, and the first layer of the muscularis propria in half of the cases. The median (range) fibrosis thickness varied from 0.87 (0.78–1.72) mm in the AM 1 group to 1.65 (1.7–1.79) mm in the control group (p = NS). Thickness of the granulation tissue varied from 0.43 (0.3–0.65) mm in the control group to 0.53 (0.25–3.94) mm in the AM 1 group (p = NS). Features of acute inflammatory infiltration were found in 10%, 60%, 40% and 60% of swine in the AM2, AM1, ES, and control groups, respectively (p = NS). The length of the neoepithelium ranged from 3.04 mm in the AM 1 group to 7.05 mm in the AM 2 group, without any statistically significant differences. Histological results are shown in table 2 and Masson's trichome-stained slides are shown in figure 3. Immunohistochemistry staining with anti-αSMA antibodies allowed for the semi-quantitative assessment of myofibroblastic activity: mean signals were 1.60±0.89 and 1.25±0.5 and mean vascular density was 2±0.71 and 2.25±0.96 (p = NS) in the AM1 and control groups, respectively. Representative findings of the immunohistochemistry staining with anti-αSMA antibody are shown on Figure 4.

**Table 2:** Histological evaluation of the esophageal wounds.

	AM 1 group≠ (n = 5)	AM 2 group# (n = 5)	ES groupα (n = 5)	Control group (n = 5)	p
Depth of the dissection					
n - (%)					
Submucosa	2–40%	2–40%	2–40%	3–60%	NS
Muscularis propria	3–60%	3–60%	3–60%	2–40%	
**Esophageal fibrosis** *	0.87 (0.78–1.72)	1.53 (1.15–2.26)	1.19 (0.28–1.95)	1.65 (0.7–1.79)	NS
**Granulation tissue** *	0.53 (0.25–0.94)	0.52 (0.23–0.79)	0.43 (0.30–2.43)	0.43 (0.30–0.65)	NS
**Acute inflammatory cell infiltrate** n - (%)	3–60%	1–20%	2–40%	3–60%	NS
**Reepithelialization** *	3.04 (2.00–5.13)	7.05 (2.23–9.28)	5.07 (2.29–10.4)	4.86 (1.23–18.9)	NS

*Median (range), in mm;

NS: non significant.

≠ : AM1 group: pigs treated with amniotic membrane and sacrificed at day 14.

# : AM2 group: pigs treated with amniotic membrane and sacrificed at symptomatic esophageal stricture occurrence.

α : ES group: pigs with esophageal stent placement alone after ESD.

**Figure 5:**
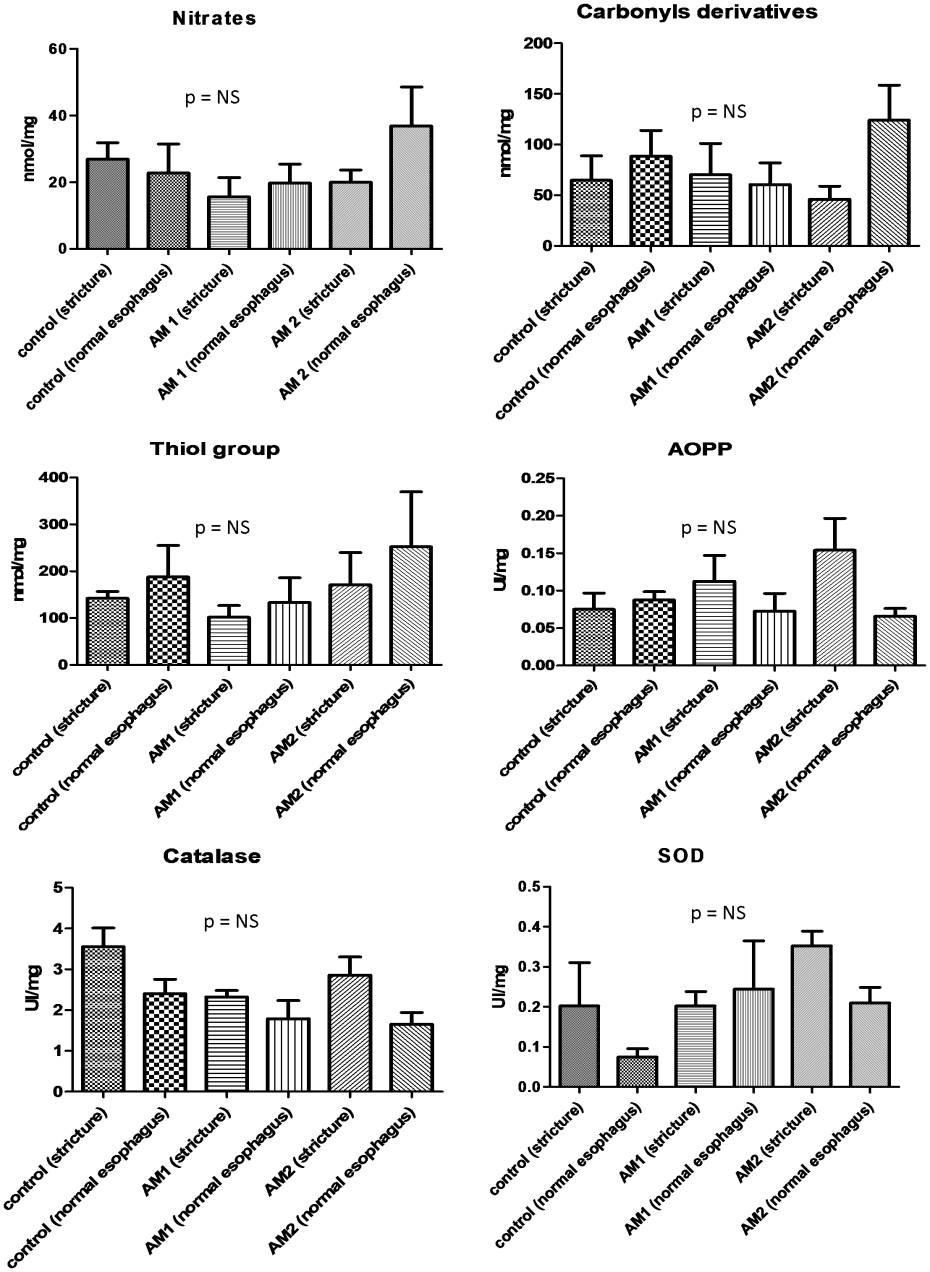
Oxidative stress markers measurements in tissue samples of strictured or normal esophagus; performed in control, AM1 and AM 2 groups.

Oxidative stress marker measurements in frozen esophageal tissue samples of strictured or normal esophagus were performed in 13 animals (4 in the AM 1 group, 5 in the AM 2, and 4 in the control group). No statistically significant differences were evidenced with regard to SOD or catalase activity, advanced oxidation proteic products, carbonyl derivatives, thiol group (total glutathione) or nitrate concentrations between the different groups. The data are presented in figure 5.

## Discussion

Esophageal stricture development after endoscopic resection can be explained by two main mechanisms:

The first is the absence of the barrier effect of the epithelium, resulting in submucosal exposure to physical or acid aggression. Epithelial growth factor injection [27], keratinocyte injection [28] or biological dressing using extracellular matrix scaffolds [23], [29], or allo/autogenic epithelial layers [30] have been used to address this problem. None of these techniques have proven to be effective in CESD. Furthermore, there are concerns about the feasibility of such techniques in routine practice, given their high costs.

The second is represented by uncontrolled inflammatory and pro-fibrotic mechanisms involved in the wound healing process, including reactive oxygen species. Anti-inflammatory medications, such as pentoxifylline and interferon alpha [31], penicillamine [32], anti-oxidants (vitamin C and E, N acetylcysteine) [33], [34], anti-fibrotic treatments, such as halofugidone [35] or corticosteroids [36], anti-mitotic drugs such as 5-fluorouracile [37] or mitomycin C [38], have been tested with insufficient results to consider their use in clinical practice. Steroid administration by local injection or by the systemic route showed promising results [10], [36]. However, other authors have reported delayed healing of esophageal ulcers, poor outcomes in terms of stricture prevention, and serious infectious adverse events related to local steroid therapy [39], and there are concerns of potential infectious adverse events, in the case of the systemic administration of corticosteroids after esophageal CESD.

We aimed to determine whether grafts of AM, a readily disposable and low cost material with previous clinical experience in humans, would be able to prevent esophageal stricture after esophageal CESD. The rationale for this hypothesis was the ability of AM to create a mechanical barrier and stimulate epithelial growth, as well as its anti-inflammatory and anti- fibrotic properties.

AM grafts allowed for a dramatic reduction of the symptomatic stricture rate on the 14^th^ postoperative day, from 100% in the control group to 33% in the AM group (p = 0.03). However, we did not evidence any statistically significant differences between AM and ES groups in terms of symptomatic stricture rates at day 14 after CESD or time of onset of a symptomatic stricture. We observed statistically significant differences in food tolerance between the AM and control groups, and a lower rate of esophageal strictures (80% vs. 100% in the AM and control groups, respectively), with a higher mean esophageal diameter (5.8±3.6 mm vs. 2.6±1.7 mm in the AM and control groups, respectively). All groups had similar CESD depth, with CESD reaching the surface of muscularis propria in almost half of the cases. Hence, histological specimens should be comparable between the groups. At day 14 after CESD, the median thickness of esophageal fibrosis in the AM 1 group was almost half that reported in the control group (0.87 mm (0.78–1.72) vs. 1.65 mm (0.7–1.79), respectively). However, swine in the AM 2 group showed major esophageal fibrosis, suggesting that the benefit of AM application was only transient. Survival time, but not AM application, impacted the nature of the granulation tissue: swine from the AM 2 group exhibited features of chronic granulation tissue (predominance of lympho-plasmocytes, low cell density) compared with swine from groups sacrificed at day 14. No differences in the thickness of the granulation tissue were evidenced. We did not observe any correlation between the depth of CESD and fibrotic or inflammatory parameters in the esophageal wall. AM did not result in higher re-epithelialization, either in groups with early sacrifice (3.04 (2.00–5.13) mm vs. 4.86 (1.23–18.9) mm for AM 1 and the control group, respectively), or in groups with late sacrifice (7.05 (2.23–9.28) mm vs. 5.07 (2.29–10.4) mm in in the AM 2 and ES groups, respectively). Only the longer survival appeared to explain the longer neo-epithelia. Immunohistochemical analysis with anti-SMA staining did not show any significant changes in myofibroblastic activity or vascular density in the treated and control groups. Also, we did not find any differences in the patterns of oxidative stress activity in the esophagus of treated and control groups.

Our study presents several limitations: firstly, endoscopic measurement of the esophageal lumen with a 7 mm biopsy forceps is imprecise and may vary with the degree of insufflation; however, estimation of the esophageal diameter on gross morphology specimens is also suboptimal, because of the retraction of tissue samples. The percentage of reduction of esophageal lumen on esophagogram [40], or the stricture index (parietal thickness/luminal diameter) [27] do not seem to be any more accurate. Esophageal diameter might be more accurately assessed on fluoroscopic images of the esophagus after opacification: however, fluoroscopy was not available on a regular basis in the setting of our study. Secondly, our swine model exhibits higher stricture rates and more severe strictures than Humans: therefore, repeated esophageal dilations (as performed in humans) are ineffective and lead to esophageal perforations. This explains the lack of data after day 35 in our study, since animals had to be sacrificed when a symptomatic stricture occurred. It must also be noted that data on the stricture rate at day 14 in similar swine models are lacking in the literature [41], [42]. Thirdly, the high rate of esophageal stent migration (despite their fastening to the esophageal wall with a clip) in a non-strictured esophagus might partly explain our results: it was not possible to determine how long the AM remained applied to the esophageal wound, and early stent migration may have jeopardized the effect of AM. Indeed, all animals with early stent migration (before day 7) had an esophageal stricture on day 14. Conversely, four out of the eight animals with the esophageal stent in place at day 7 developed an esophageal stricture a week later. Our numbers are in accordance with those of Rajan et al., who reported a stent migration rate of 67% in a similar setting [39] [39]. In a recent work, semi-covered esophageal stents were used in non-strictured esophagus, with satisfactory results in terms of stability [29]. The use of similar metallic semi-covered stents might be required in future studies.

AM grafts resulted in significant reduction of symptomatic esophageal stricture rate on the 14th postoperative day. This finding could be explained by a major reduction of the thickness of esophageal fibrosis in AM-treated swine, however not statistically significant, given the small numbers of animals in our study. However, this benefit was only temporary, and all animals developed symptomatic esophageal stricture before the 35^th^ postoperative day. Furthermore, our work did not show any statistically significant differences between swine with AM and ES placement and swine with ES placement alone. Despite the rigorous study conditions applied, we did not find any evidence in the esophagus for the anti-inflammatory, anti-angiogenic, anti-oxidant or pro-re-epithelialization properties of AM described in ophthalmology or dermatology. This may be explained by local conditions of the esophageal lumen, with chemical (acid) and mechanical (food) aggressions, and a high rate of early esophageal stent migration which occurred in almost half (7/15) of cases at day 7. Furthermore, ophthalmological studies have shown that AM grafts are usually degraded within 14 days [16], which might also explain the development of strictures past the second postoperative week. A second application of AM at day 14 might confirm this hypothesis, but would result in a cumbersome protocol. Hence, in conclusion, AM graft application does not appear to be an effective method for post-ESD stricture prevention, and further work is needed to develop a stable wound-covering agent with biological anti-fibrotic activity in this setting.
